# Efficacy of GS-441524 for Feline Infectious Peritonitis: A Systematic Review (2018–2024)

**DOI:** 10.3390/pathogens14070717

**Published:** 2025-07-19

**Authors:** Emma Gokalsing, Joana Ferrolho, Mark S. Gibson, Hugo Vilhena, Sofia Anastácio

**Affiliations:** 1 Vasco da Gama Research Centre (CIVG), Department of Veterinary Sciences, Vasco da Gama University School, Avenida José R. Sousa Fernandes 197 Lordemão, 3020-210 Coimbra, Portugal; emmagok@gmail.com (E.G.); joana.gibson@euvg.pt (J.F.); mark.gibson@euvg.pt (M.S.G.); hcrvilhena@hotmail.com (H.V.); 2 Department of Veterinary Clinics, School of Medicine and Biomedical Sciences, ICBAS-UP, University of Porto, 4050-313 Porto, Portugal; 3 Animal and Veterinary Research Centre (CECAV), University of Trás-os-Montes and Alto Douro (UTAD), Quinta de Prados, 5000-801 Vila Real, Portugal; 4 Associate Laboratory for Animal and Veterinary Science—AL4AnimalS, 1300-477 Lisboa, Portugal; 5 Center of Neurosciences and Cell Biology, Health Science Campus, 3000-548 Coimbra, Portugal; 6 University Institute of Health Sciences–CESPU (IUCS-CESPU), 4585-116 Gandra, Portugal

**Keywords:** feline infectious peritonitis, GS-441524, antiviral treatment, efficacy, remdesivir, molnupiravir, feline coronavirus

## Abstract

Feline infectious peritonitis (FIP) is a severe viral disease with a very high fatality rate. GS-441524 is an adenosine analogue that acts as an antiviral and has shown promise in FIP treatment. However, its commercialization in some regions is not yet authorized. To evaluate the efficacy of GS-441524 based on the published literature, a systematic review was conducted. This systematic review was conducted using PubMed, ScienceDirect, and Google Scholar for studies published from 2018 onwards. Following PRISMA guidelines, 11 studies (totaling 650 FIP cases treated with GS-441524 alone or in combination) were included. Therapeutic efficacy was assessed by FIP form, clinical signs, and dosage. The overall treatment success rate was 84.6%. This rate was higher when GS-441524 was combined with other antivirals and lower in cases of wet FIP or those with neurological complications. Combination therapy with other antivirals may improve outcomes in complicated FIP cases, although further studies are needed. The GS-441524 dosages associated with the best outcomes were 5–10 mg/kg once daily (or equivalent subcutaneous dose), adjusted for FIP type, severity, and presence of neurological/ocular signs. Higher dosages can be used for severe cases or to prevent relapse, but splitting into twice-daily dosing may be necessary to avoid absorption issues. In summary, this synthesis indicates that GS-441524 is a highly promising treatment for FIP, with a high success rate among treated cases. Nevertheless, randomized controlled trials are needed to establish evidence-based therapeutic protocols tailored to different FIP presentations.

## 1. Introduction

Feline infectious peritonitis (FIP) is a devastating disease caused by certain strains of feline coronavirus (FCoV) that acquire mutations leading to a virulent biotype (FIPV) [1]. Among FCoV-infected cats, only a small proportion develop FIP [2], which occurs primarily in young cats (3 months to 2 years old) and in cats from multi-cat environments like shelters or catteries, where FCoV exposure is high [2,3].

FIPV, unlike the benign feline enteric coronavirus (FECV), has a higher tropism for monocytes and macrophages, disseminating through lymphatic and vascular systems and causing a systemic disease [1]. Clinically, FIP manifests in two forms: effusive FIP, characterized by immune-mediated vasculitis and effusions in body cavities; and non-effusive FIP, characterized by granulomatous lesions in various organs. These forms can overlap. [4]. Despite being recognized since 1963, FIP has historically carried a near-100% fatality rate in cats that develop the disease [5].

**Figure 1:**
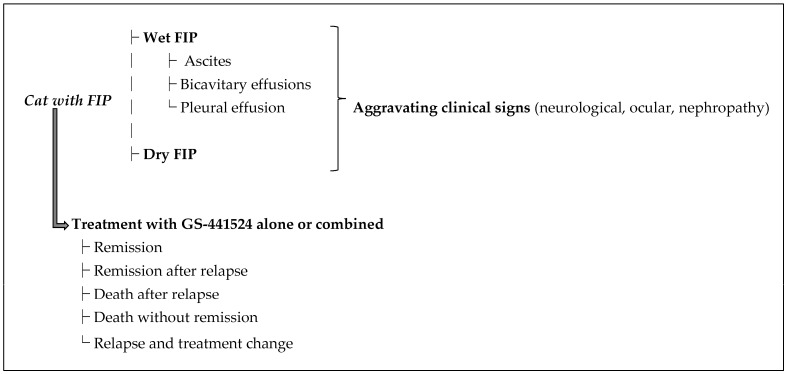
Clinical decision-making diagram for cats diagnosed with feline infectious peritonitis (FIP). Cats presenting with FIP typically exhibit either the effusive or non-effusive form of the disease. The effusive form may manifest as ascites, bicavitary effusions, or pleural effusion. Cats with either form may additionally present aggravating clinical signs, including neurological symptoms, ocular involvement, or nephropathy. Treatment options include GS-441524, alone or in combination with supportive therapies, resulting in several possible clinical outcomes: remission, remission after relapse, relapse requiring a treatment change, death after relapse, or death without remission.

Diagnosing FIP is challenging. In 2022, the AAFP/EveryCat Foundation published comprehensive FIP diagnostic guidelines [5]. A presumptive FIP diagnosis is based on the cat’s age, history, clinical signs (Figure 1), and consistent laboratory findings (e.g., cytology of effusions, hematology, and biochemistry) [2]. A confirmatory diagnosis requires a combination of the latter alongside positive PCR and immunohistochemistry [5].

Without antiviral therapy, FIP is almost invariably fatal—affected cats typically deteriorate and die or are euthanized within weeks [6]. Historically, only palliative treatments (e.g., NSAIDs, immunomodulators like interferons, and supportive care) were available, which did not cure the disease [6].

In recent years, new antiviral molecules have given hope for treating FIP. One of the most promising is GS-441524, an adenosine nucleoside analogue that inhibits the RNA-dependent RNA polymerase of coronavirus and causes premature termination of viral RNA synthesis [7]. Another antiviral that was explored is GC376, a 3C-like protease inhibitor which prevents maturation of viral polyproteins and thereby inhibits viral replication [1]. Also of note is GS-5734 (Remdesivir), the prodrug of GS-441524, which is metabolized into GS-441524 in vivo and has similar antiviral effects [8]. Molnupiravir (EIDD-2801) is yet another antiviral that has been investigated more recently for FIP, particularly as a rescue therapy in GS-441524-resistant cases [9].

Despite the success of these antivirals in research settings, GS-441524 itself is not currently approved globally as a veterinary drug. Gilead Sciences holds the patent for GS-441524 and has not authorized its veterinary use, meaning veterinarians and cat owners have resorted to black-market sources of GS-441524 in the past. They can now obtain it through licenced compounding pharmacies, which have been made available in many countries. It is legally available via veterinary “channels” in Australia, Canada, the Netherlands, the UK, and the USA as a compounded treatment. BOVA, a UK-based company supplying veterinary pharmaceuticals, supplies it in this form. The human drug Remdesivir (which is licenced for COVID-19) has been legally used as an alternative in some places (e.g., under veterinary cascade or compassionate use) [10]. The increasing use of GS-441524 in the field has dramatically improved FIP outcomes and lowered the disease’s lethality rate [7]. Nonetheless, concerns remain about the emergence of drug-resistant viral mutants, suboptimal drug distribution to certain sites (e.g., brain), and potential adverse effects of these treatments [1,11].

This review aims to systematically synthesize the literature on FIP treatment with GS-441524. Specifically, we evaluate the efficacy of GS-441524 (alone or combined with other antivirals/immunomodulators) on the survival and remission of cats with FIP, stratified by FIP form (effusive, non-effusive, mixed), presence of severe clinical signs (e.g., neurological or ocular involvement), and route of administration used.

## 2. Materials and Methods

### 2.1. Research Question

The review was guided by a research question formulated using the PICO framework (Population, Intervention, Comparison, Outcomes) [12]. In brief, Population = cats with FIP; Intervention = treatment with GS-441524 (alone or in combination); Comparison = none (single-arm studies) or between subgroups (e.g., different FIP forms or adjunct therapies); Outcome = clinical remission or survival.

### 2.2. Literature Search

A systematic literature search was conducted following the Preferred Reporting Items for Systematic Reviews and Meta-Analyses (PRISMA) 2020 guidelines [13]. We searched PubMed, ScienceDirect, and Google Scholar between 6 December 2023 and 25 January 2024 for studies published from 2018 onward (the period during which GS-441524 has been studied in FIP). The search terms included “Feline Infectious Peritonitis GS-441524 treatment”, and languages were restricted to English, Portuguese, and French. Duplicates across databases were removed. Titles and abstracts were screened to include only studies specifically evaluating GS-441524’s antiviral efficacy in naturally occurring FIP.

### 2.3. Inclusion Criteria

Studies were included if they (1) evaluated GS-441524 (alone or with other antivirals/immunomodulators) in cats with naturally occurring FIP; (2) monitored clinical outcomes (survival/remission) and reported sufficient data on FIP form, clinical signs, or treatment parameters. Both case series and individual case reports were eligible. Two studies provided additional data upon request (these are noted in the results). Studies were excluded if they were in vitro or experimental infection studies, did not actually use GS-441524 in cats, or lacked sufficient clinical detail.

### 2.4. Data Extraction

For each included study, data were extracted from the publication (and any Supplementary Material or author correspondence) on FIP form (effusive, non-effusive, mixed); effusion type in wet FIP (ascites, pleural, others); presence of aggravating clinical signs (neurological, ocular, renal) (Figure 1); GS-441524 treatment details (dosage, route of administration, duration, use of adjunct therapies); and outcomes (remission or death, including whether relapse occurred). Outcome categories were defined as follows:

- Remission: clinical recovery with no recurrence of disease after a single GS-441524 treatment course.
- Remission after relapse: recovery after an initial response, subsequent disease relapse, and then a second course of GS-441524.
- Death after relapse: initial improvement on GS-441524, but relapse occurred and the cat ultimately died or was euthanized despite additional treatment.
- Death without remission: no significant clinical improvement on GS-441524, and the cat died or was euthanized during or after the treatment course.
- Relapse with treatment change: disease relapse that was treated with a different therapy (e.g., another antiviral like Molnupiravir) due to GS-441524 failure.

Each case in each study was classified into these outcome categories (Figure 1). For clarity of analysis, we often consider “treatment success” as the sum of remission and remission after relapse (i.e., the cat survived FIP), and “treatment failure” as outcomes of death (with or without relapse).

We assessed the possibility of the duplication of cases in different studies. From those conducted in Australia, the UK, Germany, China, and the USA, we believe the large geographical separation rules out duplication in the four non-US-based studies. The two studies from the USA were conducted in California and Ohio. There is no overlap in authorship or affiliations. Th methods and results sections in these two papers confirm different age ranges and means for the enrolled animals, as well as different breeds within the cohorts. Added to the considerable physical distance between where these studies were conducted, we are satisfied there is no overlap.

Regarding the two studies published in Japan, the study published in 2021 [14] only addresses effusive FIP cases, whereas the study from 2023 [15] only addresses non-effusive and mixed FIP cases. The authors make this distinction clearly, underlining that these are separate cohorts.

Two further studies [11,16] originate from the same clinical centre; however, several details in both articles rule out case duplication. For example, the four subjects described in [11] are too young to be included in [17]. Furthermore, the combination of type of FIP, clinical signs, and treatment regimens also dismiss the possibility of duplicated cases.

### 2.5. Bias and Quality Assessment

The risk of bias in each study was assessed using the Joanna Briggs Institute (JBI) critical appraisal tools appropriate to the study design (case reports or case series) [18,19]. Common considerations included whether case inclusion criteria were clear, FIP diagnoses were definitive, all eligible cases were included, and outcomes were well described. No study was excluded based on quality, but the overall body of evidence was characterized using GRADE criteria (Grading of Recommendations Assessment, Development and Evaluation) [20]. Given that all included studies were uncontrolled case series or reports, the initial GRADE rating for each outcome was “low”. Factors such as risk of bias, inconsistency, indirectness, imprecision, and publication bias were considered to potentially downgrade confidence, whereas large effect sizes or dose–response relationships could upgrade it.

### 2.6. Data Synthesis

Data from all included studies were compiled in tables and summarized descriptively by Emma Gokalsing, working independently. Because all studies were uncontrolled and heterogeneous in design, a formal meta-analysis was not performed. Instead, we calculated pooled proportions (e.g., overall remission rates) across studies and performed subgroup analyses by stratifying cases according to:

- Treatment modality: GS-441524 alone vs. GS-441524 in combination with other antivirals (e.g., GC376, Remdesivir) or immunomodulators (e.g., interferon omega).
- FIP form: effusive vs. non-effusive vs. mixed FIP.
- Presence of aggravating signs: cases with vs. without neurological/ocular involvement.
- Route of administration: oral vs. subcutaneous, with cases that received GS-441524 by more than one route excluded.

For comparisons, outcomes were dichotomized into success *vs*. failure as defined above. Odds ratios (OR), relative risks (RR), and risk differences (RD) were computed with 95% confidence intervals (CIs) for these subgroup comparisons, mainly to explore trends rather than to derive definitive statistical conclusions (given the observational data). A chi-square test was used to assess heterogeneity in outcome distributions across subgroups, with *p* < 0.05 considered statistically significant. These calculations were done using Microsoft Excel (Microsoft Office^®^, Atlanta, GA, USA) and WinEpi epidemiological software (version 2006).

## 3. Results

### 3.1. Article Selection

The search yielded 313 unique records. After screening titles/abstracts, 45 articles remained for full-text review, and 10 additional records were identified by examining references of these articles. Ultimately, 12 studies met all inclusion criteria. One of these was excluded despite meeting criteria, because key outcome data were not extractable from the published report (even after contacting the authors). Thus, 11 studies were included in the final synthesis. The excluded study was a large retrospective study of 307 cats treated with Remdesivir/GS-441524 in the UK, which did not report individual case outcomes in a usable form. Two included studies provided supplementary data (e.g., specific outcome breakdowns) upon request, which were incorporated. The summary of the article selection process for inclusion in the study is shown in Table 1.

The 11 included studies spanned various countries and study designs, from small case series (n = 4 cats) to larger compilations (n > 100 cats). The key characteristics of each study are summarized in Supplementary Table S1 with FIP diagnosis methods also provided in Supplementary Data 1. All studies were uncontrolled (case reports or case series), and none were randomized trials. Supplementary Table S2 presents our risk-of-bias assessment for each study (no study was deemed “critical” risk; most were moderate to high risk of bias due to their observational nature). Outcome data from each study (remission, relapse, death counts) are provided in Supplementary Table S3. Data was aggregated and analyzed as described below. The overall certainty of evidence for each outcome was rated “very low” due to study design limitations, such as the fact that the included studies are uncontrolled (see Section 3.3).

### 3.2. Summary of Treated Cases and Outcomes

Across the 11 studies, data for 650 FIP cases treated with GS-441524 were compiled. Among these, 524 cases (80.6%) achieved remission with their initial GS-441524 treatment course, and an additional 26 cases (4.0%) achieved remission after experiencing a relapse and undergoing a second GS-441524 treatment. Therefore, the combined treatment success rate was 84.6% (550/650 survived in remission). Conversely, 72 cats (11.1%) died without ever reaching remission, and 2 cats (0.3%) died after initially improving and then relapsing. An additional 26 cats (4.0%) experienced a relapse and were switched to an alternative antiviral (Molnupiravir)—these cases are considered treatment failures of GS-441524 for the purpose of GS-441524 efficacy evaluation, though many of them were ultimately saved by the rescue therapy (as reported in those studies).

Among the 650 cases, 169 cats (26.0%) had what we term “complicated FIP”, defined as FIP with aggravating clinical signs such as neurological or ocular involvement. A breakdown of these cases is shown in Table 2.

Thus, in cats with any neurological and/or ocular signs, the success rate was noticeably lower (around 74.5% combined success versus ~84% in the overall population; *p* = 0.0076). Specifically, cats with only ocular complications responded as well as uncomplicated cases (around 90% success), whereas those with neurological FIP had a success rate of ~68%, and those with both neuro and ocular signs had an even lower success (~43%). These findings reflect the challenge of treating neurologic FIP.

Cases by FIP form were also categorized:

- Effusive FIP: 202 cases (31.1%). Among these, 148 (73.3%) achieved remission, 9 (4.5%) went into remission after relapse, 1 (0.5%) died after relapse, 35 (17.3%) died without remission, and 9 (4.5%) were switched to another treatment after relapse. Success rate for wet FIP was ~77.7%.
- Non-effusive FIP: 187 cases (28.8%). Outcomes: 154 (82.4%) went into remission, 10 (5.3%) went into remission after relapse, 1 (0.5%) died after relapse, 10 (5.3%) died without remission, and 12 (6.4%) switched after relapse. Success rate ~87.7%.
- Mixed FIP (both effusive and non-effusive components): 162 cases (24.9%). Outcomes: 134 (82.7%) went into remission, 3 (1.9%) went into remission after relapse, 0 died after relapse, 24 (14.8%) died without remission, and 1 (0.6%) switched after relapse. Success rate ~84.6%.

Comparing forms, non-effusive FIP cases had the highest success rate (~88%) (*p* = 0.039), significantly higher than effusive FIP (~78%). Mixed FIP was intermediate (~85%) and not significantly different from non-effusive FIP (*p* = 0.56). Because the *p*-value is higher than 0.05, more studies are needed to draw significant conclusions on this matter. Effusive FIP cases had a statistically lower success compared to non-effusive cases (*p* = 0.029). We further looked at wet FIP outcomes by effusion type when reported: cats with only ascites tended to have a slightly lower success (78.8%) than those with pleural effusion (~89%), but numbers were small and differences were not significant (*p* = 0.87). Thus, the presence of effusion (especially large-volume ascites) may correlate with lower success, but our data did not conclusively confirm effusion type as an independent prognostic factor.

Detailed data is shown on Table 3 and Table 4.

### 3.3. GS-441524 Monotherapy vs. Combination Therapy

Eight of the eleven studies (covering 551 of the 650 cats) evaluated GS-441524 as a stand-alone treatment (with only supportive care). In that combined cohort, the success rate was 83.1% (458/551) for GS-441524 monotherapy. The remaining cases in our dataset involved GS-441524 used in combination with other treatments:

- GS-441524 + Interferon omega: One study (12 cats total) reported using feline interferon-ω alongside GS-441524 in primarily dry FIP cases. Notably, all 12 cats survived (1 required an extra GS-441524 cycle) for an effective success rate of ~100% in that small sample. This regimen was associated with prolonged interferon therapy (up to 1 year) following a standard GS-441524 course [21]. Due to the small number of felines in these studies, no failure was reported in this treatment group, which means that the effectiveness of the treatment can not be assessed statistically.
- GS-441524 + Remdesivir: Two studies (37 cats) combined GS-441524 (oral) with injectable Remdesivir [8,22]. Remdesivir was either given initially for a few days or concurrently. Outcome: 35/37 survived (94.6% success). Statistically, this was not different from GS-441524 alone (*p* = 0.066) and we can not exclude that the better outcome in this drug’s combination is not due to chance. All cats with neurological signs in this group survived, but numbers were too small to obtain a small *p*-value and draw firm statistical conclusions.
- GS-441524 + GC376: Two studies (48 cats) combined GS-441524 with GC376 [9,23]. Outcome: 45/48 survived (93.8% success). Again, not statistically superior to monotherapy (*p* = 0.054). Among those with non-effusive FIP and concurrent GC376, success was 94% (including two relapses that were retreated successfully). The addition of GC376 did not clearly overcome the challenge of neurologic disease (one cat with CNS signs relapsed).

Pooling all combination-therapy cases (n = 97) yields a success rate of ~94.8%, compared to 83.1% for monotherapy. This numeric difference identifies a possible advantage to combination therapy in tough cases, but the OR and RR confidence intervals for combination vs. monotherapy crossed one (OR ~2.3 {0.8–6.7}; RR ~1.14 {0.98–1.33}; *p* = 0.11). This means that the improved outcome following combination therapy can be due to chance, and a poorer outcome is also statistically possible and plausible. For instance, it is statistically possible to obtain the opposite outcome with another case series studying combination therapy on a different group of felines with FIP. Thus, while combination regimens showed excellent outcomes, especially in small cohorts, we cannot conclusively say they are superior to GS-441524 alone given the current data. It is worth noting that no failures were reported in the interferon combination group (hence an incalculable OR for that subgroup), pointing to potential synergy that warrants further investigation (Table 5). The effectiveness of treatment is not called in question, but the superiority of one treatment over another needs further research to be assessed and draw statistically significant conclusions.

### 3.4. Influence of Clinical Factors on Efficacy

We examined the subset of complicated FIP (neurologic/ocular cases) more closely. As noted, success was lower in these cases. Among neurologic FIP cases across all studies (whether treated with higher doses or not), only 67.7% achieved success. In contrast, ocular-only cases had a 90.5% success rate, nearly as high as uncomplicated cases, indicating that standard dosing often suffices for ocular FIP. For neurologic FIP, many studies escalated GS-441524 doses (up to 10–15 mg/kg) specifically to address CNS penetration issues. Despite higher dosing, the CNS cases still had lower success, underscoring the difficulty of achieving adequate drug levels in the central nervous system. This finding aligns with the known pharmacokinetic challenge that GS-441524 faces with the blood–brain barrier [24].

It was notable that when GS-441524 was combined with other antivirals in neurologic cases, outcomes improved. In the 10 neurologic cases treated with GS-441524 + Remdesivir, all survived. In eight neurologic cases treated with GS-441524 + GC376, seven survived (one died, despite the combination) [25]. These small numbers are promising, though a lot more data is needed to determine whether combination therapy may improve outcomes for neurologic FIP.

Regarding the FIP form, we found that effusive FIP cases had worse outcomes than non-effusive FIP. When controlling for other factors, effusive FIP had an OR of ~0.49 for success compared to non-effusive FIP, and an RR indicating about an 11% reduction in success probability. This supports clinical observations that non-effusive FIP tends to respond better to treatment than effusive FIP, perhaps because cats with effusive FIP often have more advanced disease or a higher virus burden. Mixed FIP cases (with partial effusion) had outcomes closer to non-effusive FIP. For this limited number of cases, if effusions were small or localized, the prognosis with GS-441524 remained good.

GS-441524 was given through single or multiple routes of administration, with the majority of cases being single-route oral (522/650) or subcutaneous (95/650). Regarding the oral route, 454/522 cats (87%) achieved remission, whilst the proportion was 73/95 (77%) when delivered subcutaneously. This difference (87% vs. 77%) is statistically significant (*p* = 0.01); however, the two groups differ in size considerably and are not stratified by the type of FIP or the treatment regimen. The absolute numbers of cases for some of these parameters are too small to apply statistics (single animals in some instances). Therefore, we cannot conclude with confidence that delivery via the oral route represents an intrinsically more effective method than subcutaneous delivery. A larger, stratified cohort is needed to evaluate this effectively.

### 3.5. Safety and Adverse Effects

While efficacy was the primary focus, the included studies also reported on safety. No life-threatening adverse effects of GS-441524 were noted in any study. The most common issue was discomfort at injection sites for subcutaneous administration, which was universally reported when injections were used (often manifesting as pain or localized swelling) [11]. Oral GS-441524 was generally well tolerated; mild elevations in liver enzymes and mild lymphocytosis were occasionally observed but did not usually necessitate stopping treatment [26]. One report documented two cats developing uroliths (bladder stones) composed of GS-441524 or its metabolites during treatment—an unusual side effect thought to result from high drug concentrations in urine [27]. Those cases underscore the importance of monitoring during treatment, especially if high doses are used or if the cat has urinary tract symptoms.

Combination therapy did not appear to introduce significant new safety concerns in the limited cases reported. Remdesivir injections can cause transient renal enzyme elevations and injection-site phlebitis in some cases, but in the FIP studies reviewed, it was used for a relatively short duration and was well tolerated [28]. GC376 is known to cause reversible teeth discoloration in juvenile animals (a concern in lab studies) and occasional injection-site reactions; no severe issues were reported in the FIP cats on GC376, aside from one case of facial skin ulceration that may have been related to the drug or the disease itself [25]. Interferon omega can cause transient fever or malaise, but cats in those combination studies did not have significant adverse reactions reported [29].

It should be noted that although compounded medication containing GS-441524 is available in some markets, GS-441524 is not legally available everywhere, leading many owners to procure it from unregulated sources. Recent analyses of such black-market products found that some contain incorrect concentrations of GS-441524 or even different antivirals like molnupiravir mislabeled as GS-441524 [30]. Some formulations had very low pH [31], causing severe injection site damage [29]. These findings highlight a broader safety issue: the need for globally available quality-controlled, approved formulations to ensure safe treatment. Globally legalizing and regulating GS-441524 (or its prodrug) for veterinary use would mitigate these risks.

### 3.6. Certainty of Evidence

Using the GRADE criteria, we rated the overall certainty of the evidence from this review as low to very low (Supplementary Table S4). This is mainly because all included data are from uncontrolled case series without randomization or blinding, making them susceptible to various biases. There was also some inconsistency (success rates varied across studies, though generally high) and imprecision (wide confidence intervals in subgroup analyses, especially for small subgroups like high-dose cases). On the other hand, the therapeutic effect observed (over 80% survival from a disease that is otherwise almost 100% fatal) is striking and consistent enough to be convincing, even if formally “low certainty.” We emphasize that while the results suggest GS-441524 is effective, the absence of controlled trials means caution should be taken when drawing definitive conclusions about comparative efficacy (e.g., combination vs. monotherapy, or optimal dosing). Randomized trials would greatly increase confidence in findings and help establish causality.

## 4. Discussion

In this systematic review, we compiled and analyzed the published evidence on treating FIP with GS-441524. The results indicate that GS-441524 is efficacious, transforming FIP from a fatal disease into a treatable condition for the majority of affected cats. Across 650 reported cases, approximately 85% survived and achieved remission with GS-441524 therapy, which is a striking outcome considering the historical near-100% mortality of FIP.

Our analysis confirms several clinical impressions and provides some new insights:

High Overall Efficacy: GS-441524, as a sole therapy, yielded about an 83% success rate in pooled data. When including combination treatments, success exceeded 90% in some cohorts. These findings concur with earlier reports of ~80–90% cure rates in field use of GS-441524 or its prodrugs [32,33]. By comparison, historically, no treatment reliably induced remission in FIP, so this represents a significant improvement.

Effusive vs. Non-Effusive FIP: Cats with non-effusive (dry) FIP responded somewhat better than those with effusive FIP, which aligns with clinical experience. Effusive FIP is the more acute fulminant form with a shorter clinical course, and extensive immune-complex deposition, which may be harder to fully reverse, even with antivirals. Additionally, large-volume effusions can be caused by a high viral load and severe inflammation. Still, it is notable that even in effusive FIP, roughly 78% of cats survived—again, a vast improvement on 0%.

Neurological/Ocular FIP: Cats with CNS involvement remain the most challenging to cure. The blood–brain barrier limits the penetration of GS-441524; even at higher doses, drug levels in cerebrospinal fluid are much lower than in plasma. This likely explains why standard dosing is often insufficient for neuro-FIP. Clinicians now often use ~10 mg/kg for neuro cases (and some advocate even higher or twice-daily dosing) [16]. Alternative strategies, such as using more lipophilic analogues or combination therapy (e.g., adding drugs that cross into the CNS), might further improve neuro-FIP outcomes. The success seen with Molnupiravir in rescue cases is encouraging—Molnupiravir is orally administered and can penetrate the CNS. Combining GS-441524 with Molnupiravir or other CNS-penetrant antivirals may be a fruitful approach in the future.

Combination Therapy: While our analysis did not prove a significant advantage to combining GS-441524 with other antivirals, the numerically higher survival in those cohorts and the particular success in some tough cases (e.g., all neuro cases on GS + Remdesivir survived) identify this strategy as promising in certain scenarios. It is logical that two antivirals targeting different viral proteins (polymerase and protease, for instance) could have additive or synergistic effects. Moreover, Remdesivir essentially delivers the same active molecule but via a different route—some protocols start with Remdesivir injections to rapidly achieve high blood levels and then switch to oral GS-441524 for convenience [8]. Australian veterinarians have adopted Remdesivir (since it is legally obtainable) followed by oral GS-441524, with reported outcomes similar to those in this review (an approximately 90% cure rate) [10]. Interferon omega’s role is less clear; it has immunomodulatory effects and might help in controlling secondary infections or modulating the immune response. In a couple of small studies, adjunct interferon seemed to help, but those were not controlled comparisons. As a minimum, we can say that interferon was not detrimental, and it could be considered as an adjunct, especially in non-effusive FIP cases [21].

Treatment Duration: Most studies treated for at least 12 weeks, which has become the standard recommendation (this duration was originally extrapolated from the first experimental trial of GS-441524) [24]. A few reports experimented with shorter courses (e.g., 8 weeks) or longer ones (beyond 12 weeks). Anecdotally, shorter courses have higher relapse rates [11]. One recent study suggests that even a 6-week course might suffice for some mild cases, but relapse remains a risk [34]. In general, 12 weeks is still advised, and if there is any residual disease at 12 weeks, extending treatment or increasing the dose for a few extra weeks is recommended [16]. Our review found that some cats required a second round of treatment after a relapse—often at a higher dose or for a longer period—to fully cure the disease. As a safety margin, some practitioners treat for 2 weeks beyond the resolution of clinical signs to ensure clearance of the virus. In our dataset, about 4% of cats relapsed after the first round and were then successfully retreated; thus, monitoring after the initial treatment period is crucial.

Relapses and Resistance: A small number of cats did not respond or relapsed repeatedly. Viral RNA from some of these refractory cases has shown mutations in the virus’s polymerase or spike proteins, potentially conferring partial resistance to GS-441524 [9]. Molnupiravir has been used as a “rescue” in these cases, as it has a different mechanism (causing lethal mutagenesis of the virus). Roy et al. (2022) reported that 8/11 cats who failed with GS-441524 treatment achieved remission when switched to Molnupiravir [9]. This highlights the importance of having multiple antiviral options available, especially as wider use of GS-441524 could exert selective pressure on the virus. The combination or sequential use of GS-441524 and Molnupiravir is an active area of investigation.

Safety and Side Effects: GS-441524 has a wide therapeutic margin in cats; most adverse effects are localized or mild. The occurrence of urolithiasis in two cases is intriguing—it may be that GS-441524 (or a metabolite) can crystallize in urine in some circumstances. Ensuring cats stay well-hydrated during treatment might be a reasonable precaution, especially at high doses. The injection pain issue has largely been mitigated by the move to oral formulations. Many owners now prefer to use oral GS-441524 from day one (to avoid subjecting the cat to daily painful injections) [35]. In situations where oral absorption might be compromised (e.g., a cat with vomiting or inappetence), initial injectable dosing can be performed and then transitioned to oral once the cat is stable [10]. Overall, the tolerability of GS-441524 is excellent compared to many chemotherapeutics or antiviral drugs used in other species.

Treating FIP with GS-441524 offers new hope, yet animal welfare considerations must remain central. While many cats experience remission, treatment failures can occur, especially with under-dosing, advanced disease, or drug resistance. Adverse effects, such as injection site pain, vomiting, or liver enzyme elevation, require careful monitoring. Furthermore, reliance on grey-market sources raises ethical and safety concerns due to unregulated drug quality. Ensuring informed consent, adequate veterinary oversight, and access to humane alternatives is crucial. A balanced approach respects both the drive to save lives and the responsibility to avoid undue suffering during experimental or costly interventions.

Regulatory Status and Access: Despite the compelling evidence of efficacy, GS-441524 is only approved in certain countries, and supplied as a compounded pharmaceutical. Remdesivir, which is chemically very similar, is now legal for veterinary FIP treatment in Australia, Canada, the UK, and some EU countries. It is being used off-label by veterinarians in the USA as a legal workaround [10]. The FDA’s guidance in 2022 opened the door for compounding Remdesivir or similar drugs for FIP under certain conditions [10]. The Federation of Veterinarians of Europe has also advocated for legal pathways to access these life-saving treatments [10]. In Europe, the use of extemporaneous products containing GS-441524 has now been made possible by some manufacturers, giving a legal and safer chance of survival for FIP patients [36]. There is no common regulatory standard between countries, which makes it difficult even for clinicians in the field to understand what treatment options are legally available in their country. For this reason, veterinarians might not even consider GS-441524 or its analogues as a treatment option for FIP.

Limitations: The main limitations of this review stem from the available studies. All data were from uncontrolled trials; hence, improvements in cats could theoretically have been influenced by ancillary care or spontaneous recovery (though spontaneous recovery from FIP is exceedingly rare). Controlled randomized studies are needed to confirm the efficacity of GS-441524 in the treatment of FIP to increase the certainty level of any conclusion on the matter and to draw clear and practical guidelines on the use of the antiviral in FIP patients. Currently, the data available is not statistically robust enough to allow veterinarians to implement rules and guidance in clinical decision-making. For instance, no statistically based conclusion can be drawn on whether neurological FIP should be treated with combination therapy or a monotherapy, or which dosage should be used and for how long. The conclusions on this matter are based on experience and not evidence, due to the absence of controlled randomized studies. Publication bias is likely—veterinarians or owners who had great success might be more inclined to publish than those with failures. However, given the consistent reports from various independent groups, the efficacy signal is strong. We also had to rely on pooled data that combine different dosing regimens and case mixes. There is inherent heterogeneity: for example, one study might focus on CNS cases on high doses, while another was mostly uncomplicated cases on standard doses—comparing results between them is not straightforward. Wherever possible, we stratified data to make “apples to apples” comparisons.

Another limitation important to highlight is that the age of the felines we analyzed was not always available. We were therefore unable to include this parameter as part of our study. We acknowledge this may be a significant factor in the response to treatment. The literature does not yet contain multiple and consistent examples of age being a significant factor affecting GS-441524 efficacy when treating effusive vs. non-effusive FIP. In one of the studies we analyzed [11], a field trial involving 31 cats, with ages 3.4 to 73 months (approx. 0.3–6 years), had 26 effusive and 5 non-effusive cases. Older cats and non-effusive cats responded as well as young cats and those with effusive FIP. But this is just one example, and clearly more studies are needed to assess this issue adequately.

Five of the eleven included studies used gold standard methodologies, with a further five using a thorough range of techniques, including PCR. Only one study [9] lacked adequate details, with cats presumed to have FIP based on prior veterinary diagnosis, but no details are provided. As a further limitation of our review, it may be possible that some of these cases were not actually FIP, though as a cohort of just 26 cases, and for each, the outcome defined as “relapse and change treatment”, they did not impact upon the data we calculated for remission or death.

Future Directions: What the field needs now are controlled randomized clinical trials. A randomized trial of GS-441524 (or its prodrug) at different doses would help determine the minimum effective dose and confirm that the outcomes are truly as good as they appear. Trials comparing GS-441524 monotherapy vs. combination (e.g., with an adjunct like GC376 or Molnupiravir) would be valuable, particularly for neurologic FIP. Additionally, long-term follow-up of treated cats is needed to ensure there are no late relapses or chronic issues. Thus far, reports of cats cured of FIP show they live normal lives and even successfully receive vaccinations or deal with other illnesses without FIP recurrence [11].

In conclusion, this review provides a comprehensive update on the use of GS-441524 in FIP. The evidence, albeit from uncontrolled studies, consistently shows a high efficacy in treating a disease once considered a death sentence. This has already translated into thousands of lives saved worldwide through underground efforts. As the veterinary community moves towards globally legitimizing these treatments, the knowledge from these studies will inform protocols and help optimize outcomes. With continued research, we hope to refine dosing for special cases (like neurologic FIP), manage drug resistance, and perhaps one day integrate FIP antivirals into official treatment guidelines. What has been achieved so far is a strong step forward for feline medicine—turning FIP into a curable disease in most instances—and ongoing research and advocacy are poised to further improve and formalize FIP treatment.

## 5. Conclusions

FIP has historically been one of the most dreaded feline diseases, but the advent of antiviral therapy (in particular GS-441524 and its analogues) has dramatically improved the prognosis. This review confirms that GS-441524 is a highly effective treatment for FIP, inducing remission in the majority of cases, including both effusive and non-effusive forms. The appropriate dosage typically lies in the 4–10 mg/kg range once daily for at least 12 weeks, with higher doses (or more frequent dosing) required for cases with CNS involvement. In our analysis, remission from uncomplicated FIP correlated with lower doses and straightforward protocols, whereas complicated cases (especially neurological FIP) benefit from more aggressive treatment strategies, with combination therapy a promising option.

Treatment combinations (GS-441524 with interferon omega, Remdesivir, or GC376) appear safe and potentially helpful for difficult cases, though randomized studies are needed to determine if they provide a significant advantage. Cases that fail initial GS-441524 therapy can often be rescued with Molnupiravir as an alternative antiviral, highlighting the importance of having multiple treatment options available.

All evidence indicates that GS-441524 is generally well tolerated by cats. The main side effects to watch for are injection site reactions (largely mitigated by using oral formulations) and, rarely, potential issues like drug-related uroliths. Practitioners should also be aware of the varying quality of black-market products and counsel pet owners on the risks. The global legalization and regulation of FIP antivirals would greatly enhance treatment safety and accessibility and eliminate the ethical quandaries some veterinarians face.

In summary, based on the compiled literature, GS-441524 is an effective therapy for FIP, converting a once-hopeless disease into one with a good chance of cure. To fully realize the benefits for all cats, further research (especially controlled clinical trials) is necessary to refine treatment regimens and to validate long-term outcomes. The collective experience so far provides a strong foundation for developing evidence-based treatment guidelines for FIP in the near future. With continued advancements, the goal is to establish FIP as a fully manageable condition, tailoring therapy to each cat’s disease presentation and ensuring that every cat with FIP has access to life-saving treatment.

Another future consideration is antiviral resistance. Widespread use of a single antiviral could lead to resistant FCoV strains. Monitoring and rotating or combining therapies may become important. It may be prudent to treat the most severe cases with dual therapy from the start to prevent the emergence of resistant variants, analogous to how combination therapy is used in HIV and other viral infections to suppress resistance.

## Figures and Tables

**Table 1:** Summary of the number of articles considered at each stage of the selection process for inclusion in the study. This table presents the number of references retrieved, screened, and finally selected from each database during the literature review process.

	PubMed Central	Google Scholar	Science Direct	Cross-Reference
References retrieved	182	102	29	-
References selected after abstract screening and duplicate removal	36	7	2	10
References selected * after full-text reading	11	0	0	0

* The 11 selected references were also found in other databases besides PubMed Central but were excluded from those due to duplication during the screening process.

**Table 2:** Number of cases of complicated FIP divided into subgroups of reported clinical signs and treatment outcomes.

	Number of Cases	Remission	Remission After Relapse	Death After Relapse	Relapse Requiring Treatment Change
Total	169	116	10	2	13
Neurological signs	106	66	7	2	9
Ocular signs	48	42	1	0	1
Neurological and ocular	7	2	1	0	3
Renal	8	6	1	0	0

**Table 3:** Odds ratio, relative risk, and risk difference for the efficacy of treatment in complicated FIP compared to non-complicated FIP, with a 95% confidence interval.

Comparison—Efficacy of Treatment in Complicated vs. Non-Complicated FIP	OR [95% CI]	RR [95% CI]	RD [95% CI]	*p* Value
Overall Efficacy	0.39 [0.25–0.61]	0.85 [0.77–0.93]	−0.14 [−0.22; −0.07]	<0.0001
Efficacy of GS-441524 Alone	0.40 [0.25–0.61]	0.84 [0.77–0.93]	−0.14 [−0.22; −0.06]	<0.0001
Efficacy of GS-441524 with Interferon omega	Not estimable	Not estimable	Not estimable	n.a.
Efficacy of GS-441524 with Remdesivir	Not estimable	Not estimable	0.07 [0.02–0.11]	n.a.
Efficacy of GS-441524 with GC376	0.09 [0.007–1.13]	0.80 [0.56–1.14]	−0.20 [−0.48; 0.08]	0.0281

**Table 4:** Odds ratio, relative risk, and risk difference for the efficacy of treatment in wet FIP compared to mixed and dry FIP, with a 95% confidence interval.

Comparison—Efficacy of Treatment in Effusive FIP vs.	OR [95% CI]	RR [95% CI]	RD [95% CI]	*p* Value
GS-441524 Monotherapy in Mixed FIP	0.64 [0.37–1.1]	0.94 [0.85–1.04]	−0.05 [−0.13; 0.03]	0.0996
GS-441524 Monotherapy in Non-effusive FIP	0.49 [0.28–0.85]	0.89 [0.81–0.97]	−0.10 [−0.17; −0.03]	0.0096

**Table 5:** Odds ratio, relative risk, and risk difference for the efficacy of GS-441524 in combination therapy compared to GS-441524 monotherapy, with a 95% confidence interval.

Comparison—Efficacy of GS-441524 Combined with:	OR [95% CI]	RR [95% CI]	RD [95% CI]	*p* Value
Interferon omega	Not estimable	1.20 [1.16–1.25]	0.17 [0.14–0.20]	n.a. ^a^
Remdesivir	3.55 [0.8–15.0]	1.14 [1.04–1.23]	0.11 [0.02–0.18]	0.0664
GC376	3.05 [0.9–10.0]	1.13 [1.03–1.22]	0.11 [0.03–0.17]	0.0542

^a^ n.a.—not applicable.

## Data Availability

Data are contained within the article.

## Supplementary Materials

The following supporting information can be downloaded at: https://www.mdpi.com/article/10.3390/pathogens14070717/s1, Table S1: Summary of selected published studies reporting treatment outcomes in cats diagnosed with feline infectious peritonitis (FIP). Table S2: Assessment of risk of bias in selected studies on feline infectious peritonitis (FIP) using the Joanna Briggs Institute (JBI) critical appraisal tool. Table S3: Summary of clinical outcomes in cats with feline infectious peritonitis (FIP) treated with GS-441524, remdesivir, and other antivirals, as reported in selected studies. Table S4: Summary of evidence and certainty levels (GRADE) regarding the efficacy of GS-441524-based treatments for feline infectious peritonitis (FIP).

## Abbreviations

The following abbreviations are used in this manuscript:

3CLPRO	3-Cysteine-like Protein
EFCOV	Enteric Feline Coronavirus
EMA	European Medicines Agency
FCOV	Feline Coronavirus
FDA	Food and Drug Administration
FeLV	Feline Leukemia Virus
FIP	Feline Infectious Peritonitis
FIPV	Feline Infectious Peritonitis Virus
FIV	Feline Immunodeficiency Virus
GRADE	Grating of Recommendations, Assessment, Development, and Evaluation
MPRO	Main Protein
Nsp5	Non-structural Protein 5
Nsps	Non-structural Proteins
OR	Odds Ratio
PICO	Population, Interventions, Comparisons, Outcomes
PRISMA	Preferred Reporting Items for Systematic Reviews and Meta-Analysis
RD	Risk Difference
RR	Relative Risk
RT-qPCR	Reverse Transcription-Quantitative Polymerase Chain Reaction
