# Supplementary material for: Efficacy of GS-441524 for Feline Infectious Peritonitis: A Systematic Review (2018–2024)
Source: Pathogens. 2025 Jul 19;14(7):717. doi: 10.3390/pathogens14070717 (PMC12298711; doi:10.3390/pathogens14070717)
Supplement: Supplementary file 1 [file pathogens-14-00717-s001.zip › Table S1.pdf]

Supplementary Table S1. *Summary of selected published studies reporting treatment outcomes in cats diagnosed with feline infectious peritonitis (FIP).*

The table lists key references by author, country of study, number of cases treated, therapeutic approach (including GS-441524, Remdesivir, Interferon-omega, GC376, and Molnupiravir), and reported outcomes such as remission, relapse, or death.

Selected References	Country	Characteristics
Addie, D., Silveira, C., Aston, C., Brauckmann, P., Covell-Ritchie, J., Felstead, C., Fosbery, M., Gibbins, C., Macaulay, K., McMurrough, J ., Pattison, E., & Robertson, E. (2022). Alpha-1 acid glycoprotein reduction differentiated recovery from remission in a small cohort of cats treated for feline infectious peritonitis. Viruses, 14(4), 744. https://doi.org/10.3390/v14040744	UK	12 cases: treatment with oral GS-441524 and Interferon-omega<br>12 remissions
Coggins, S., Norris, J.M., Malík, R., Govendir, M., Hall, E., Kimble, B., & Thompson, M.N. (2023). Outcomes of treatment of cats with feline infectious peritonitis using parenterally administered remdesivir, with or without transition to orally administered GS-441524. Journal of Veterinary Internal Medicine, 37(5), 1772–1783. https://doi.org/10.1111/jvim.16803	Australia	13 cases: treatment with oral Remdesivir and oral GS-441524<br>12 remissions; 1 death
Cosaro, E., Pires, J., Castillo, D., Murphy, BG, & Reagan, KL (2023). Efficacy of Oral Remdesivir Compared to GS-441524 for Treatment of Cats with Naturally Occurring Effusive Feline Infectious Peritonitis: A Blinded, Non-Inferiority Study. Viruses, 15(8), 1680. https://doi.org/10.3390/v15081680	USA	9 cases: treatment with oral GS-441524<br>5 remissions; 4 deaths
Dickinson, P.J., Bannasch, M.J., Thomasy, S.M., Murthy, V.D., Vernau, K.M., Liepnieks, M., Montgomery, E., Knickelbein, K.E., Murphy, B.G., & Pedersen, N.C. (2020). Antiviral treatment using the adenosine nucleoside analogue GS-441524 in cats with clinically diagnosed neurological feline infectious peritonitis. Journal of Veterinary Internal Medicine, 34(4), 1587–1593. https://doi.org/10.1111/jvim.15780	USA	4 cases: treatment with subcutaneous GS-441524<br>2 remissions; 1 remission after relapse; 1 death after relapse
Green, J., Syme, H. M., & Tayler, S. (2023). Thirty-two cats with effusive or non-effusive feline infectious peritonitis treated with a combination of remdesivir and GS-441524. Journal of Veterinary Internal Medicine, 37(5), 1784–1793. https://doi.org/10.1111/jvim.16804	UK	26 cases: 24 cases of treatment with injectable Remdesivir (intravenous, subcutaneous) and oral GS-441524, 2 cases with oral GS-441524<br>23 remissions; 1 remission after relapse; 2 deaths without remission
Katayama, M., & Uemura, Y. (2023b). Prognostic prediction for therapeutic effects of mutian on 324 Client-Owned cats with feline infectious peritonitis based on clinical laboratory indicators and physical signs. Veterinary Sciences, 10(2), 136 .https://doi.org/10.3390/vetsci10020136	Japan	324 cases: treatment with oral GS-441524<br>279 remissions; 11 remissions after relapse; 34 deaths without remission
Katayama, M., & Uemura, Y. (2021). Therapeutic effects of Mutian® Xraphconn on 141 Client-Owned cats with feline infectious peritonitis predicted by total bilirubin levels. Veterinary Sciences, 8(12), 328. https://doi.org/10.3390/vetsci8120328	Japan	141 cases: treatment with oral GS-441524<br>113 remissions, 3 remissions after relapse, 25 deaths without remission

Krentz, D., Zenger, K., Alberer, M., Felten, S., Bergmann, M., Dorsch, R., Matiasek, K., Kolberg, L., Hofmann-Lehmann, R., Meli, M.L. , Spiri, A.M., Horak, J., Weber, S., Holicki, C.M., Groschup, M.H., Zablotski, Y., Lescrinier, E., Koletzko, B., Von Both, U., & Hartmann, K. (2021). Curing Cats with Feline Infectious Peritonitis with Oral Multi-Component Drug Containing GS-441524. Viruses, 13(11), 2228. https://doi.org/10.3390/v13112228	Germany	18 cases: treatment with oral GS-441524<br>18 remissions
Lv, J., Yang, B., Wang, Y., Yang, L., Jin, Y., & Dong, J. (2022). Effect of GS-441524 in combination with the 3C-like protease inhibitor GC376 on the treatment of naturally transmitted feline infectious peritonitis. Frontiers in Veterinary Science,9. https://doi.org/10.3389/fvets.2022.1002488	China	46 cases: treatment with subcutaneous GS-441524 and subcutaneous GC376<br>43 remissions, 2 remission after relapse, 1 death without remission
Pedersen, N.C., Perron, M., Bannasch, M.J., Montgomery, E., Murakami, E., Liepnieks, M., & Liu, H. (2019). Efficacy and safety of the nucleoside analog GS-441524 for treatment of cats with naturally occurring feline infectious peritonitis. Journal of Feline Medicine and Surgery, 21(4), 271-281. https://doi.org/10.1177/1098612x19825701	USA	31 cases: treatment with subcutaneous GS-441524<br>18 remissions, 7 remissions after relapse, 1 death after relapse, 5 deaths without remission
Roy, M., Jacque, N., Novicoff, W. M., Li, E., Negash, R., & Evans, S. (2022). Unlicensed Molnupiravir is an Effective Rescue Treatment Following Failure of Unlicensed GS-441524-like Therapy for Cats with Suspected Feline Infectious Peritonitis. Pathogens, 11(10), 1209. https://doi.org/10.3390/pathogens11101209	USA	26 cases: treatment with oral or subcutaneous GS-441524, 22 cases as single treatment, 2 cases in combination with GC376, 2 cases in combination with GC376 and Molnupiravir<br>26 relapse and change of treatment
