# Supplementary material for: Efficacy of GS-441524 for Feline Infectious Peritonitis: A Systematic Review (2018–2024)
Source: Pathogens. 2025 Jul 19;14(7):717. doi: 10.3390/pathogens14070717 (PMC12298711; doi:10.3390/pathogens14070717)
Supplement: Supplementary file 1 [file pathogens-14-00717-s001.zip › Table S2.pdf]

Supplemetary Table S2. *Assessment of risk of bias in selected studies on feline infectious peritonitis (FIP) using the Joanna Briggs Institute (JBI) critical appraisal tool.*

The table summarizes the methodological limitations identified in each study, highlighting specific biases detected and the overall categorization of risk of bias (low, moderate, or high). This assessment supports the interpretation of the strength of the evidence presented in each reference.

Evaluated reference	Bias detected with the JBI tool	Categorization of risk of bias
Addie, D., Covell-Ritchie, J., Jarrett, O., & Fosbery, M. (2020). Rapid resolution of Non-Effusive feline infectious peritonitis uveitis with oral adenosine nucleoside analogue and feline interferon omega. Viruses, 12(11), 1216. https://doi.org/10.3390/v12111216	No adverse effects detected	Low
Addie, D., Silveira, C., Aston, C., Brauckmann, P., Covell-Ritchie, J., Felstead, C., Fosbery, M., Gibbins, C., Macaulay, K., McMurrough, J ., Pattison, E., & Robertson, E. (2022). Alpha-1 acid glycoprotein reduction differentiated recovery from remission in a small cohort of cats treated for feline infectious peritonitis. Viruses, 14(4), 744. https://doi.org/10.3390/v14040744	Integrity of case inclusion doubtful, lack of animal demographic data	Moderate to high
Coggins, S., Norris, J.M., Malík, R., Govendir, M., Hall, E., Kimble, B., & Thompson, M.N. (2023). Outcomes of treatment of cats with feline infectious peritonitis using parenterally administered remdesivir, with or without transition to orally administered GS-441524. Journal of Veterinary Internal Medicine, 37(5), 1772–1783. https://doi.org/10.1111/jvim.16803	/	Low
Cosaro, E., Pires, J., Castillo, D., Murphy, BG, & Reagan, KL (2023). Efficacy of Oral Remdesivir Compared to GS-441524 for Treatment of Cats with Naturally Occurring Effusive Feline Infectious Peritonitis: A Blinded, Non-Inferiority Study. Viruses, 15(8), 1680. https://doi.org/10.3390/v15081680	Doubtfully complete and consecutive inclusion	Moderate to high
Dickinson, P.J., Bannasch, M.J., Thomasy, S.M., Murthy, V.D., Vernau, K.M., Liepnieks, M., Montgomery, E., Knickelbein, K.E., Murphy, B.G., & Pedersen, N.C. (2020). Antiviral treatment using the adenosine nucleoside analogue GS-441524 in cats with clinically diagnosed neurological feline infectious peritonitis. Journal of Veterinary Internal Medicine, 34(4), 1587–1593. https://doi.org/10.1111/jvim.15780	Very succinct past history, CFov titration absent in one of the 4 cases	Moderate
Green, J., Syme, H. M., & Tayler, S. (2023). Thirty-two cats with effusive or non-effusive feline infectious peritonitis treated with a combination of remdesivir and GS-441524. Journal of Veterinary Internal Medicine, 37(5), 1784–1793. https://doi.org/10.1111/jvim.16804	CFov research desirable but not essential for inclusion	Moderate
Katayama, M., & Uemura, Y. (2023b). Prognostic prediction for therapeutic effects of mutian on 324 Client-Owned cats with feline infectious peritonitis based on clinical laboratory indicators and physical signs. Veterinary Sciences, 10(2), 136 .https://doi.org/10.3390/vetsci10020136	Inclusion criteria not detailed	Moderate
Katayama, M., & Uemura, Y. (2021). Therapeutic effects of Mutian® Xraphconn on 141 Client-Owned cats with feline infectious peritonitis predicted by total bilirubin levels. Veterinary Sciences, 8(12), 328. https://doi.org/10.3390/vetsci8120328	Inclusion criteria not detailed	Moderate
Krentz, D., Zenger, K., Alberer, M., Felten, S., Bergmann, M., Dorsch, R., Matiasek, K., Kolberg, L., Hofmann-Lehmann, R., Meli, M.L. , Spiri, A.M., Horak, J., Weber, S., Holicki, C.M., Groschup, M.H., Zablotski, Y., Lescrinier, E., Koletzko, B., Von Both, U., & Hartmann, K. (2021). Curing Cats with Feline Infectious Peritonitis with Oral Multi-Component Drug Containing GS-441524. Viruses, 13(11), 2228. https://doi.org/10.3390/v13112228	Doubtfully consecutive inclusion, little clinic demographic information	Low to moderate
Lv, J., Yang, B., Wang, Y., Yang, L., Jin, Y., & Dong, J. (2022). Effect of GS-441524 in combination with the 3C-like protease inhibitor GC376 on the treatment of naturally transmitted feline infectious peritonitis. Frontiers in Veterinary Science,9. https://doi.org/10.3389/fvets.2022.1002488	Inclusion criteria not detailed	Moderate
Pedersen, N.C., Perron, M., Bannasch, M.J., Montgomery, E., Murakami, E., Liepnieks, M., & Liu, H. (2019). Efficacy and safety of the nucleoside analog GS-441524 for treatment of cats with naturally occurring feline infectious peritonitis. Journal of Feline Medicine and Surgery, 21(4), 271-281. https://doi.org/10.1177/1098612x19825701	------	Low
Roy, M., Jacque, N., Novicoff, W. M., Li, E., Negash, R., & Evans, S. (2022). Unlicensed Molnupiravir is an Effective Rescue Treatment Following Failure of Unlicensed GS-441524-like Therapy for Cats with Suspected Feline Infectious Peritonitis. Pathogens, 11(10), 1209. https://doi.org/10.3390/pathogens11101209	Dosage used unknown, means of diagnosis unknown	High
