# Supplementary material for: Efficacy of GS-441524 for Feline Infectious Peritonitis: A Systematic Review (2018–2024)
Source: Pathogens. 2025 Jul 19;14(7):717. doi: 10.3390/pathogens14070717 (PMC12298711; doi:10.3390/pathogens14070717)
Supplement: Supplementary file 1 [file pathogens-14-00717-s001.zip › Table S3.pdf]

Supplementary Table S3. *Summary of clinical outcomes in cats with feline infectious peritonitis (FIP) treated with GS-441524, remdesivir, and other antivirals, as reported in selected studies.*
The table presents the number and type of FIP cases (dry, wet, or mixed), presence of aggravating clinical signs (e.g., neurological, ocular, renal), treatment protocols (including duration and route of administration), and remission rates. Treatment responses vary according to clinical form and severity, with higher remission rates generally observed in non-neurological cases treated with GS-441524 for extended periods.

Selected References	Number of Cases	Aggravating Clinical Signs	Treatment	Evolution
Addie, D., Covell-Ritchie, J., Jarrett, O., & Fosbery, M. (2020). Rapid resolution of Non-Effusive feline infectious peritonitis uveitis with oral adenosine nucleoside analogue and feline interferon omega. Viruses, 12(11), 1216. https://doi.org/10.3390/v12111216	1 dry PIF	Ocular	GS-441524 oral 50d IF-omega after	100% remission
Addie, D., Silveira, C., Aston, C., Brauckmann, P., Covell-Ritchie, J., Felstead, C., Fosbery, M., Gibbins, C., Macaulay, K., McMurrough, J ., Pattison, E., & Robertson, E. (2022). Alpha-1 acid glycoprotein reduction differentiated recovery from remission in a small cohort of cats treated for feline infectious peritonitis. Viruses, 14(4), 744. https://doi.org/10.3390/v14040744	4 dry PIF	--	GS-441524 oral 55d [29-84d] IF-omega after	100% remission
	5 wet PIF	1 neurological	GS-441524 oral 73d [37-117d] IF-omega after	100% remission
	2 mixed PIF	--	GS-441524 oral 51d [50-52d] IF-omega after	--
Coggins, S., Norris, J.M., Malík, R., Govendir, M., Hall, E., Kimble, B., & Thompson, M.N. (2023). Outcomes of treatment of cats with feline infectious peritonitis using parenterally administered remdesivir, with or without transition to orally administered GS-441524. Journal of Veterinary Internal Medicine, 37(5), 1772–1783. https://doi.org/10.1111/jvim.16803	1 dry PIF	--	Remdesivir SC and GS-441524 oral 84d	100% remission
	6 wet PIF	--	Remdesivir SC and GS-441524 oral >84d	83.3% remission
Cosaro, E., Pires, J., Castillo, D., Murphy, BG, & Reagan, KL (2023). Efficacy of Oral Remdesivir Compared to GS-441524 for Treatment of Cats with Naturally Occurring Effusive Feline Infectious Peritonitis: A Blinded, Non-Inferiority Study. Viruses, 15(8), 1680. https://doi.org/10.3390/v15081680	9 wet PIF	--	GS-441524 oral 84d	55% remission
Dickinson, P.J., Bannasch, M.J., Thomasy, S.M., Murthy, V.D., Vernau, K.M., Liepnieks, M., Montgomery, E., Knickelbein, K.E., Murphy, B.G., & Pedersen, N.C. (2020). Antiviral treatment using the adenosine nucleoside analogue GS-441524 in cats with clinically diagnosed neurological feline infectious peritonitis. Journal of Veterinary Internal Medicine, 34(4), 1587–1593. https://doi.org/10.1111/jvim.15780	4 dry PIF	4 neurological and 3 ocular	GS-441524 SC 129.5d [98-189d]	75% remission
Green, J., Syme, H. M., & Tayler, S. (2023). Thirty-two cats with effusive or non-effusive feline infectious peritonitis treated with a combination of remdesivir and GS-441524. Journal of Veterinary Internal Medicine, 37(5), 1784–1793. https://doi.org/10.1111/jvim.16804	5 dry PIF	2 neurological and 1 ocular	Remdesivir IV/SC 4.2d [0-6d] and oral GS-441524 80d [78-84d]	100% remission
	21 wet PIF	3 ocular and 1 neurological	Remdesivir IV/SC 15.3d [0-73d] and oral GS-441524 61.3d [2-79d]	90% remission
Katayama, M., & Uemura, Y. (2023b). Prognostic prediction for therapeutic effects of	163 dry PIF	43 neurological and 30 ocular	GS-441524 oral >84d	94% remission

mutian on 324 Client-Owned cats with feline infectious peritonitis based on clinical laboratory indicators and physical signs. Veterinary Sciences, 10(2), 136 .https://doi.org/10.3390/vetsci10020136	161 mixed PIF	46 neurological and 11 ocular	GS-441524 oral >84d	85% remission
Katayama, M., & Uemura, Y. (2021). Therapeutic effects of Mutian® Xraphconn on 141 Client-Owned cats with feline infectious peritonitis predicted by total bilirubin levels. Veterinary Sciences, 8(12), 328. https://doi.org/10.3390/vetsci8120328	141 wet PIF	--	GS-441524 oral >84d	82% remission
Krentz, D., Zenger, K., Alberer, M., Felten, S., Bergmann, M., Dorsch, R., Matiasek, K., Kolberg, L., Hofmann-Lehmann, R., Meli, M.L. , Spiri, A.M., Horak, J., Weber, S., Holicki, C.M., Groschup, M.H., Zablotski, Y., Lescrinier, E., Koletzko, B., Von Both, U., & Hartmann, K. (2021). Curing Cats with Feline Infectious Peritonitis with Oral Multi-Component Drug Containing GS-441524. Viruses, 13(11), 2228. https://doi.org/10.3390/v13112228	2 dry PIF	1 neurological and 2 ocular	GS-441524 oral >84d	100% remission
	16 wet PIF	--	GS-441524 oral >84d	90% remission
Lv, J., Yang, B., Wang, Y., Yang, L., Jin, Y., & Dong, J. (2022). Effect of GS-441524 in combination with the 3C-like protease inhibitor GC376 on the treatment of naturally transmitted feline infectious peritonitis. Frontiers in Veterinary Science,9. https://doi.org/10.3389/fvets.2022.1002488	10 dry PIF	8 renal	GS-441524 SC and GC376 SC 28d	90% remission
	36 wet PIF	--	GS-441524 SC and GC376 SC 28d	97.2% remission
Pedersen, N.C., Perron, M., Bannasch, M.J., Montgomery, E., Murakami, E., Liepnieks, M., & Liu, H. (2019). Efficacy and safety of the nucleoside analog GS-441524 for treatment of cats with naturally occurring feline infectious peritonitis. Journal of Feline Medicine and Surgery, 21(4), 271-281. https://doi.org/10.1177/1098612x19825701	5 dry PIF	--	GS-441524 SC >84d	100% remission
	26 wet PIF	3 neurological	GS-441524 SC >84d	76.9% remission
Roy, M., Jacque, N., Novicoff, W. M., Li, E., Negash, R., & Evans, S. (2022). Unlicensed Molnupiravir is an Effective Rescue Treatment Following Failure of Unlicensed GS-441524-like Therapy for Cats with Suspected Feline Infectious Peritonitis. Pathogens, 11(10), 1209. https://doi.org/10.3390/pathogens11101209	15 dry PIF	8 neurological and 3 ocular	GS-441524 oral >84d	0% remission
	16 wet PIF	4 neurological and 1 ocular	GS-441524 oral >84d	0% remission
	1 mixed PIF	1 neurological	GS-441524 SC >84d	0% remission
