# Supplementary material for: Efficacy of GS-441524 for Feline Infectious Peritonitis: A Systematic Review (2018–2024)
Source: Pathogens. 2025 Jul 19;14(7):717. doi: 10.3390/pathogens14070717 (PMC12298711; doi:10.3390/pathogens14070717)
Supplement: Supplementary file 1 [file pathogens-14-00717-s001.zip › Table S4.pdf]

Supplementary Table S4. *Summary of evidence and certainty levels (GRADE) regarding the efficacy of GS-441524-based treatments for feline infectious peritonitis (FIP).*

This table summarizes the synthesized evidence on treatment efficacy, the associated level of certainty according to the GRADE approach, the references supporting each statement, and the factors influencing the level of certainty.

Evidence	GRADE Certainty Level	References Used in Synthesis	Factors Influencing Certainty Level
**GS-441524 is effective as monotherapy in 83.1% of cases**	Low	[9], [11], [19], {25], [30], [33], [34], [35]	Detected risk of bias in the included references
**GS-441524 monotherapy is less effective in wet FIP than in mixed FIP**	Very low	[9], [11], [19], {25], [30], [33], [34], [35]	Detected risk of bias in the references, imprecision (95% CI)
**GS-441524 monotherapy is less effective in wet FIP than in dry FIP**	Low	[9], [11], [19], {25], [30], [33], [34], [35]	Detected risk of bias in the included references
**GS-441524 monotherapy is effective in 67% of complicated cases with aggravating signs**	Very low	[9], [11], [19], {25], [30], [33], [34], [35]	Detected risk of bias in the references, lack of information
**Doses between 5–10 mg/kg of GS-441524 are more effective than higher doses (SC or equivalent oral)**	Very low	[11], [19], {25], [30], [33], [34], [35]	Detected risk of bias, indirect comparisons, imprecision (95% CI, few animals treated with >10 mg/kg)
**GS-441524 with Interferon-omega after treatment cycle is effective and possibly more effective than monotherapy**	Very low	[9], [11], [17], [18], [19], {25], [30], [33], [34], [35]	Detected risk of bias, intervention differences (treatment duration), imprecision (few animals treated with the combination)
**GS-441524 is effective in combination with Remdesivir and possibly more effective than monotherapy**	Low	[8], [9], [11], [19], {25], [30], [33], [34], [35]	Detected risk of bias in the included references
**GS-441524 is effective in combination with GC376 and possibly more effective than monotherapy**	Very low	[9], [11], [19], [20], {25], [30], [33], [34], [35]	Detected risk of bias, intervention differences (treatment duration)

Note: A dose-response gradient associated with animals presenting aggravating signs or relapses was a factor influencing all evidence levels.
